# The early response during the interaction of fungal phytopathogen and host plant

**DOI:** 10.1098/rsob.170057

**Published:** 2017-05-03

**Authors:** Yilin Shen, Na Liu, Chuang Li, Xin Wang, Xiaomeng Xu, Wan Chen, Guozhen Xing, Wenming Zheng

**Affiliations:** State Key Laboratory of Wheat and Maize Crop Science/Collaborative Innovation Center of Henan Grain Crops, College of Life Science, Henan Agricultural University, Zhengzhou 450002, People's Republic of China

**Keywords:** fungal pathogens, host plant, early infection, molecular interactions, avirulent genes, resistance genes

## Abstract

Plants can be infected by a variety of pathogens, most of which can cause severe economic losses. The plants resist the invasion of pathogens via the innate or acquired immune system for surviving biotic stress. The associations between plants and pathogens are sophisticated beyond imaging and the interactions between them can occur at a very early stage after their touching each other. A number of researchers in the past decade have shown that many biochemical events appeared even as early as 5 min after their touching for plant disease resistance response. The early molecular interactions of plants and pathogens are likely to involve protein phosphorylation, ion fluxes, reactive oxygen species (ROS) and other signalling transduction. Here, we reviewed the recent progress in the study for molecular interaction response of fungal pathogens and host plant at the early infection stage, which included many economically important crop fungal pathogens such as cereal rust fungi, tomato *Cladosporium fulvum*, rice blast and so on. By dissecting the earlier infection stage of the diseases, the avirulent/virulent genes of pathogen or resistance genes of plant could be defined more clearly and accurately, which would undoubtedly facilitate fungal pathogenesis study and resistant crop breeding.

## 1. Introduction

One of the differences between plants and animals is that plants are unable to move. They rely on the immune system to perceive and identify a pathogen, and then make a series of response mechanisms [1–3]. Most interactions between plants and pathogens start from genetic and molecular aspects. The ‘gene-for-gene’ hypothesis was first proposed by Harold Henry Flor via investigating flax and flax rust race-specific resistance in 1955 [4]. The biochemical basis of this hypothesis is the interaction between resistance (*R*) gene products and avirulence (*Avr*) gene products. Plants have developed multiple mechanisms to recognize pathogen invasion and trigger immune responses directly or indirectly [5].

So far, it has been reported that dozens of plant diseases have been caused by pathogenic interaction systems. Many studies have shown that the interactions between plants and pathogens associated with protein phosphorylation. Phosphatases and protein kinases play a vital role in the activation of the early-stage disease resistance responses [6,7]. Rapid phosphorylation response to pathogens and other elicitors includes some downstream mitogen-activated protein kinases (MAPKs) [8–11], calmodulin protein kinases [12] and syntaxin-like proteins [13].

Knowledge of phosphorylation events and their regulation is crucial to understand the mechanisms of plant and pathogen interactions [14]. Protein phosphorylation, a common regulation mode *in vivo*, plays an important role in cellular signal transduction process. Phosphorylation of proteins occurs mainly on serine (included threonine) and tyrosine, and the enzymes and functions of these two types of amino acids are different.

A decade ago, plant innate immune response regulatory mechanisms were analysed via quantitative phosphoproteomic [15]. The interactions occurred earlier than researchers thought from past research into the response of fungal pathogen–host interaction. Benschop *et al*. [16] found that the earliest signalling events triggered by elicitor occurred within minutes in *Arabidopsis* through quantitative phosphoproteomics analysis. The same study provided a novel insight about plant defence signal transduction and early interaction response.

So far, although many reports about the early plant–pathogen interaction research have been published, there is not a clear definition about the ‘early stage’ of interaction. Here, we define the early interaction period as the time point before a pathogen completes the invasion of a host plant (usually within 24 h after pathogen and plants begin to contact each other). In recent years, with the development of proteomics, genomics and transcriptomics techniques, more and more early host–pathogen interaction studies were undertaken from large-scale phosphoproteomics [14,16,17], genomics and transcriptomics, such as genomic analysis of *Fusarium graminearum* and wheat during early stages [18], maize–*Colletotrichum graminicola* early transcriptional events analysis [19], *Medicago truncatula–Verticillium* wilt transcriptomic study of early root responses [20], de novo transcriptome analysis of *Zoysia japonica* and *Rhizoctonia solani* in early invasion [21], comparative transcriptomics analysis of the rice varieties Digu and Lijiangxintuanheigu (LTH) and *Magnaporthe oryzae* [22]. Although the early plant–pathogen interaction has been studied extensively, most of the early molecular events that occur in host and pathogen are largely unknown. Early interactions manifestly play an important role in the further research of disease-resistance mechanisms and signalling pathways. Hunting the plant-resistant or pathogen-avirulent genes involved as early as possible in the interaction process will underpin the theoretical basis for disease resistance research and crop genetic improvement.

## 2. The early responses of host plants to pathogens

‘Immunity’ is a protective reaction in which the organism maintains their own physiological balance and stability by identifying and eliminating antigenicity foreign body. The plant immune system formed two divergent branches in the long-term evolution and development process: the sophisticated and specific adaptive immunity and the more universal innate immunity. The adaptive immune system can specifically recognize and selectively remove invading pathogens. However, it would take several weeks to form a sustained response and the majority of organisms lack this acquired immune system [23,24]. Compared with the adaptive immune system, the innate immune system does not need specialized immune cells to develop a protective response [24].

**Figure 1.**
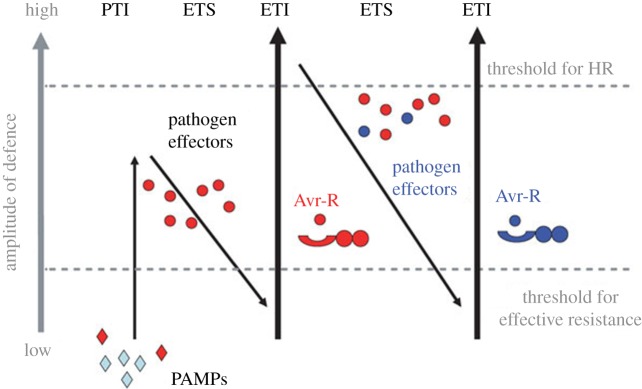
A zigzag model of the plant immune system [28]. PTI: PAMP-triggered immunity; ETS: effector-triggered susceptibility; ETI: effector-triggered immunity; PAMPs: pathogen-associated molecular patterns; HR: hypersensitive cell death response. This model can be divided into four stages. In phase 1, plants recognize PAMPs via PRRs that triggered PTI. In phase 2, effectors of invading pathogens lead to ETS or interfere with PTI. In phase 3, an NB-LRR protein specifically recognizes a pathogen effector (indicated in red) directly or indirectly, resulting in ETI. The resistance or HR induced by ETI is faster and stronger than PTI. In phase 4, natural selection perhaps forms new effectors through horizontal gene flow (in blue) replacing the old effectors (in red), and plants generate new *R* genes to resist pathogens, resulting in ETI again.

The innate immune system, on the other hand, involves a population of cells and signalling pathways that constitutively function to respond rapidly to pathogens at the site of infection [24,25]. The cells of the innate immune system detect pathogen-associated molecular patterns (PAMPs) and microbe-associated molecular patterns (MAMPs) via their pattern recognition receptors (PRRs) [24,26]. PRRs include NOD-like receptors (NLRs), Toll-like receptors (TLRs), RIG-I-like receptors (RLRs) and C-type lectin receptors (CLRs) [27]. The plant has formed two kinds of innate immune mechanism—PAMP-triggered immunity (PTI) and effector-triggered immunity (ETI)—that lead to a rapid disease response in the process of long-term cooperative coevolution with pathogen [28–31]. The two branches of the plant immune system can be described as a ‘zigzag’ model (figure 1) [28]. ETI was first discovered in plants that are dependent on the plant resistance proteins (R proteins) to identify pathogen-secreted proteins directly or indirectly and activate a strong resistance reaction inhibiting pathogen infection [24]. PTI is a relatively weak resistance reaction activated by PRR identifying conservative pathogen PAMPs [32]. Currently, the molecular mechanisms of PTI and ETI defence response have been deeply investigated in *Arabidopsis thaliana* [33]. The immune system is the foundation of the interaction between fungi and host.

Some scholars have found that the early response of crops' cells or tissues was triggered by elicitors. For example, fungal elicitor specifically induces the proteins transient, rapid and consecutive phosphorylation in the parsley (*Petroselinum crispum*) cells, and the phosphorylation activates some pathogen resistance-related genes. In the microsome and part of the cytoplasm, a neutral 45 kDa protein phosphorylated as early as 1 min after being treated with elicitor and the increase of a 26 kDa nuclear protein phosphorylation starts also at the earliest stage [34].

## 3. The signal transduction processes in the early response

The initial touching of pathogen and plant would rapidly trigger the signal transduction process on the plasma membrane and cytoplasm of plant cells [35]. The involved signal transduction in the early response covered many pivotal channels which can set subsequent responses at a multi-level of gene expression patterns. Among them, the signal components of ion flux, salicylic acid (SA) and other hormones were mostly investigated in recent reports [35–37].

The phosphorylation reaction was associated with the presence of Ca^2+^ involved in the signal transduction processes [34]. Protein phosphorylation events occurred *in vivo* within minutes when elicitor treated tomato cells. The function of elicitor was completely blocked by the protein kinase inhibitors K-252a and staurosporine. The protein kinase inhibitors can also inhibit the early biochemical responses induced by elicitors [38].

The ion flux events of plant responses to MAMPs occurred within approximately 0.5–2 min [39,40]. These changes include increased influx of Ca^2+^ and efflux of K^+^, and an efflux of anions, particularly of nitrate [41]. The ion fluxes lead to membrane depolarization [42]. Even though little is known about the ion channels, MAMPs were evident to stimulate an influx of Ca^2+^ from the apoplast and caused a rapid increase of Ca^2+^concentrations in cytoplasm, which might activate calcium-dependent protein kinases [43].

Plant hormones could play a critical role in plant defence against pathogens. Salicylic acid (SA) and ethylene can induce the expression of *defensing like* (*DEFL*) genes in *A. thaliana* [44,45]. The SA and jasmonic acid (JA) defence pathways can interact synergistically or antagonistically depending on the faced pathogens [46–49]. Plant stomata are barriers against microbial infection. PAMPs can trigger stomata closure in SA-dependent manner. Virulence factor COR from *Pst* DC3000 could inhibit the PAMP-induced abscisic-acid (ABA) signalling in the guard cell [50]. SA and fungal elicitors (α-elicitin and β-elicitin) could together rapidly activate a 48 kDa SA-induced protein kinase (SIPK) in tobacco [10].

## 4. Plant disease resistance genes involved in the early response

A few important studies of host resistance genes were initiated at early infection stage (table 1). The milestone report for the early interaction was published in 2011 for barley stem rust [51]. It was a devastating disease caused by *Puccinia graminis* f. sp. *tritici* (*Pgt*) for barley production in most areas of North America until the barley cultivars (cvs.) with *Rpg1* gene were first announced in 1942. Henceforth, *Rpg1* gene has protected barley cultivars from severe stem rust losses for over 70 years. The *Rpg1* located in the short arm of barley chromosome 1(7H) is a novel resistance gene homology with receptor kinases [58–60]. The highly susceptible cultivar Golden Promise became a high resistance disease due to transformed the *Rpg1* gene by genetic engineering [61]. Although the resistance of the *Rpg1* gene is wide, it cannot function to all virulent types of *Pgt* [62]. The *Rpg1* gene encodes a constitutively expressed protein containing two tandem kinase domains: the protein kinase 1 (pK1) domain and protein kinase 2 (pK2) domain. The pK1 is a pseudokinase, whereas the pK2 domain is catalytically active, and both domains are required for stem rust resistance. The pseudokinase pK1 domain is associated with disease resistance and the pK2 domain is involved in protein phosphorylation [63,64].

**Table 1.** Related plants genes response to pathogen at early plant–pathogen interaction. hpi: hours post inoculation; gene: the gene of host plant involved in the early interaction significantly.

plant	gene	hpi	pathogen
barley	*Rpg1*	5 min	*Puccinia graminis* f. sp. *tritici* (*Pgt*) [51]
tomato	*Cf-9*	5 min	*Cladosporium fulvum* [12]
maize	*ClCUT7*	3	*Curvularia lunata* [52]
rice	*OsAAE3*	12	*Magnaporthe oryzae* [53]
rice	receptor kinases (RKs) genes	5, 10, 20	*M. oryzae* [22]
wheat	*TaCDPK2*	4	*P. triticina* (*Pt*) [54]
wheat	*TaCAMTA4*	4	*Pt* [55]
wheat	*Lr57*	12	*Pt* [56]
wheat	*TaCERK1*, *TaCEBiP*, *TaRboh*	24	*P. striiformis* f. sp. *tritici* (*Pst*) [57]

**Figure 2.**
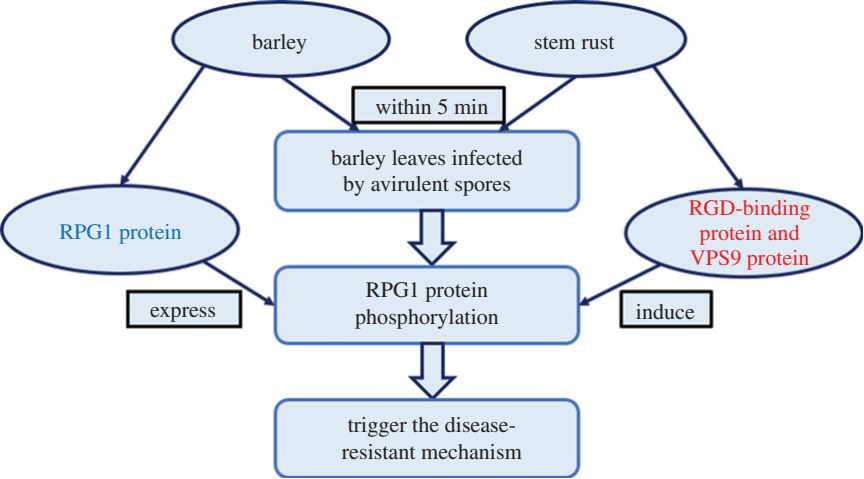
The flow chart of the interaction between stem rust fungus and barley. Barley with *Rpg1* resistance gene inoculated with avirulent and viable stem rust fungal pathotype of *Puccinia graminis* f. sp. *tritici* MCCF triggered disease resistance responses. The RPG1 protein (in blue) constitutively expressed in host cells can be phosphorylated within 5 min by the interaction between *Rpg1* gene product and RGD-binding protein and VPS9 protein (in red) in stem rust. The phosphorylation triggers a series of signalling pathways and the resistance mechanism in barley.

The RPG1 protein is a functional kinase located in the plasma membrane, endomembranes and cytosol. The resistance protein RPG1 disappeared rapidly (within 5 min) when barley seedling leaves were inoculated by avirulent and viable stem rust fungus pathotype MCCF (figure 2). The disappearance of the RPG1 protein is due to phosphorylation and the phosphorylated status sustained for 20 h after inoculation. It is suggested that RPG1 protein phosphorylation is essential for disease resistance. The reciprocal responses of barley and stem rust belong to ETI of plant. Based on these observations, it is speculated that there would be a unique mechanism for pathogen recognition and signalling in barley that we do not know yet [5,63,64].

After the ‘gene-for-gene’ theory was put forward, a number of novel *Avr* genes and homologous *R* genes have been identified. The fifirst fungal *Avr* gene (*Avr9* gene) was reported in 1991 via molecular cloning [29,65]. Several kinase genes were reported to be involved in the pathogen–host interactions. SA-induced protein kinase (SIPK) and wounding-induced protein kinase (WIPK), activated by tobacco mosaic virus (TMV) in tobacco, are members of the mitogen-activated protein (MAP) kinase family. Activation of WIPK depends on the disease-resistance gene *N* and phosphorylation of tyrosine and serine/threonine. SIPK activation was involved in resistance responses and tyrosine phosphorylation [9,10,66].

The protein encoded by the *Cf-9* gene has resistance to the fungus *Cladosporium fulvum* in tomato, and the corresponding avirulence gene is *Avr9*. *Cf-9* gene encodes a membrane-anchored extracytoplasmic glycoprotein including 27 leucine-rich repeats (LRRs). By dissecting the *Cf-9* gene, Tina Romeis *et al*. [11] identified the 46 kDa and 48 kDa protein kinases similiar to WIPK and SIPK. The activation of both kinases achieved through post-translational mechanisms, and the process of the activation is involved in Ca^2+^ influx and tyrosine phosphorylation [11,67–70]. A membrane-bound, calcium-dependent protein kinase (CDPK) removed from 68 to 70 kDa within 5 min after being treated with Avr9 elicitor. Avr9/Cf-9 activated a CDPK via a phosphorylation event [12]. Protein phosphorylation was necessary for many early Avr9/Cf-9-signalling events, and the race-specific signalling pathway was involved in three kinds of kinases: the two mitogen-activated protein kinases (MAPK), WIPK and SIKP, and the calcium-dependent protein kinase NtCDPK2. Both Avr9 and Cf-9 induced a tobacco 32 kDa syntaxin phosphorylation rapid and transient, leading to reactive oxygen species production. Syntaxin phosphorylation and NtSyp121 transcript levels increased at 24 h, which were triggered by the race-specific elicitor Avr9, but not by race-specific elicitor flg22^P.aer^ [13].

In the incompatible combination of wheat and leaf rust (Lovrin 10 and leaf rust race 260), the expression of the *TaCDPK2* gene was obviously increased at the levels of mRNA and protein while the *TaCAMTA4* gene expression level started to decrease gradually after wheat leaves inoculated leaf rust after 4 h. This result suggested that the *TaCDPK2* gene and the *TaCAMTA4* gene were involved in the interaction between wheat and leaf rust, and had a positive or negative regulation to disease resistance in wheat [54,55].

Cutinase has various functions, such as eliciting host derived signals and fungal spore attachment. The expression of the *ClCUT7* gene (from a cutinase gene family) upregulated at 3 h after maize inoculated *Curvularia lunata.* That cutinase may play a role in early fungal–plant interactions [52].

In order to find possibly related resistance genes in peanut and avirulence genes in stem rot fungus, Jogi *et al.* [71] selected four peanut cultivars (A100-32, Georgia Green, GA-07 W and York) with increasing resistance levels and a virulent S*. rolfsii* strain to study the early plant–pathogen interaction. Finally, the first 454 sequencing was performed at 4 days after peanut inoculation of S*. rolfsii.* Further studies of possibly related resistance genes and avirulence genes would be useful to the research of early host–pathogen interaction.

The early interactions of *blast fungus* (*M. oryzae*) and rice occur at the apoplast [72]. Liu *et al.* identified an AMPBP *OsAAE3* gene from rice through the early interaction between rice and *M. oryzae* [22]*.* The *OsAAE3* gene is located in cytoplasm, and it is expressed in all tissues of rice. The *Os AAE3* gene is homologous to Arabidopsis *AAE3*, and it encodes a 4-coumarate-Co-A ligase (4CL) like protein. *OsAAE3* over-expression leads to programmed cell death, decreased fertility rate of anther and inhibition of the floret development. In brief, the *OsAAE3* gene is a negative regulator in rice blast resistance [53]. Li *et al*. [22] identified 48 receptor kinases (RKs) genes in Digu via comparative transcriptomics analysis of the rice variety Digu (durably resistant) and LTH (susceptible) inoculated by *M. oryzae* (5, 10 and 20 hpi). Their study reveals that membrane-associated RKs play significant roles in the early response to *M. oryzae*. An elicitor-responsive gene *EL2* expresses rapidly and instantaneously when the leaves or roots of rice are treated with the elicitor *N*-acetylchitooligosaccharide within 30 min. The hyphal growth of rice blast fungus was remarkably delayed but not inhibited on account of the elicitor infecting the rice seedling. PR-1 and PR-10 (PBZ1), the disease resistance genes, were triggered systematically and locally by the elicitor although the defence response mechanism in rice is not clear [73].

## 5. Pathogen genes involved in the early response

Very few studies and discoveries were published on fungal pathogen genes involved in the early interaction (table 2). Also, the mechanisms of some known avirulent genes (e.g. stem rust genes: the *RGD*-binding gene and the *VPS9* gene) have been unknown.

**Table 2.** Pathogen genes involved in the early response to plant. hpi: hours post inoculation; gene: the genes of fungal phytopathogen involved in the early interaction significantly.

pathogen	gene	hpi	interaction plant
*Puccinia graminis* f. sp. *tritici*	*RGD*-binding gene, *VPS9* gene	5 min	barley [51]
*Cladosporium fulvum*	*Avr9*	3–5 min	tomato [11,67]
*Phytophthora sojae*	*Avr1b*	24	soya bean [74]
*Colletotrichum higginsianum*	*ChMK1*	no clear	cruciferous crops [75]
*Magnaporthe oryzae*	*MGS0074*, *MGS0274*, *MGS0338*, *MGS0718*, *MGS0997*, *MGS1242*, and *MGS1460* in Y99-63	24	rice [76]

In their research of molecular interaction between stem rust and barley, Nirmala *et al*. found that the arginine-glycine-aspartic acid peptide loops can prevent the formation of adhesion structures for spore attachment, the germination of the dynamic spores and the phosphorylation of RPG1 [51]. They purified and identified two proteins: arginine-glycine-aspartic acid (RGD)-binding protein with fibronectin type III and breast cancer type 1 susceptibility protein domains, and vacuolar protein sorting-associated protein 9 (VPS9 protein) with a coupling of ubiquitin to endoplasmic reticulum degradation domain from the ungerminated avirulent rust spores via the arginine-glycine-aspartic acid affinity chromatography. RGD-binding protein and VPS9 protein together induce hypersensitive response (HR) *in vivo*, phosphorylation and degradation of RPG1 in barley with a functional *Rpg1* gene. The *RGD*-binding gene and the *VPS9* gene are constitutively expressed in almost all cells of the avirulent race of stem rust fungus MCCF [58]. So far, the understanding of these two genes is not comprehensive. There is still a lot of work to do describing their functional network.

The avirulent gene *Avr9* of tomato leaf mildew is another disease-related gene involved in the early interaction. The research results for *Avr9* genes was initially compared even with those of *VPS9* gene and *RGD*-binding gene of rust fungus. *Avr9* gene encodes a preprotein that contains 63 amino acids which could function during the interaction between the fungus *Cladosporium fulvum* and tomato. Avr9 generated K^+^ outward-rectifying by 2.5-fold to threefold and almost completely suppressed inward-rectifying of K^+^ within 3–5 min. The K^+^ channel reactions were specific and irreversible [11,68].

Another important research was reported for oomycetes. Shan *et al.* first cloned the avirulence gene *Avr1b* of oomycete pathogen *Phytophthora sojae* by finestructure genetic mapping [74]. The *Avr1b* gene contains two genes: the *Avr1b*-1 gene and the *Avr1b*-2 gene. The *Avr1b*-1 gene was localized to a single 60 kb bacterial artificial chromosome (BAC). The *Avr1b*-1 gene is polymorphic and encodes a small, hydrophilic secreted protein that is a specific elicitor. The Avr1b-1 protein triggered a specific and systemic HR in soya bean leaves (carrying the *Rps*1b resistance gene). Avr1b-1 protein entering the soya bean leaf cells need RXLR (Arg-X-Leu-Arg,×is any amino acid) and dEER (Asp-Glu-Glu-Arg) [77,78]. Experiments revealed that the *Avr1b*-2 gene required the accumulation of the mRNA of the *Avr1b*-1 gene. That is to say that *Avr1b*-2 controlled the accumulation of *Avr1b*-1 mRNA. One of the advantages of the *Avr1b*-1 gene is that it could cause the hypersensitive response to spread to the whole plant. But its maximum expression was 24 h and 48 h after inoculation. It is clear that *Avr1b*-1 gene works later than *Avr9* gene, *VPS9* gene and *RGD*-binding gene. And early researches of this gene are not clear [74].

In the interaction between *Colletotrichum higginsianum* and cruciferous crops, Wei *et al.* investigated a Fus3/Kss1-related MAPK gene (*ChMK1*) from *Colletotrichum higginsianum* [75]*.* The ChMK1 is essential to pathogenicity, appressorium formation, conidiation production, cell wall integrity, growth rate and melanin formation for *C. higginsianum.* That is to say *ChMK1* gene plays an essential role in the early pathogen infection.

This kind of early response also could be affected by nutrition limitation [76]. The expression of seven genes (*MGS0074*, *MGS0274*, *MGS0338*, *MGS0718*, *MGS0997*, *MGS1242* and *MGS1460*) in Y99-63 (one strain of rice blast fungus), encoding cysteine-rich proteins, were upregulated to different extents in the early *M. oryzae*–rice interaction under nitrogen limitation. Cysteine-rich proteins might be enrolled in the cross-talking of nitrogen limitation and the early infection response [76].

## 6. Conclusion and discussion

Here, we reviewed the recent progress in the study for molecular interaction response of fungal pathogens and host plant at the early infection stage, which included some economically important crop fungal pathogens such as cereal rust fungi, tomato *Cladosporium fulvum*, *M. oryzae* and so on. According to the research so far, the distinct mechanisms for the early molecular interactions of plants and pathogens are likely to involve protein phosphorylation, ion fluxes, reactive oxygen species (ROS) and other signalling transduction.

The barley leaves start responses–protein phosphorylation and then trigger the disease resistance mechanism within 5 min of inoculating the stem rust pathogen avirulent urediniospores MCCF. During the interaction between tomato and leaf mildew, the product of the fungus *Cladosporium fulvum* avirulence gene *Avr9* resulted in K^+^ salt loss by 2.5-fold to threefold and almost complete suppression of K^+^ salt within 3–5 min*.* The *Rpg1* gene and the *Cf-9* gene can resist the invasion of the *VPS9* gene, the *RGD*-binding gene and the *Avr9* gene rapidly via the trigger of disease-resistant mechanisms. The examples of these two interactions showed that plants and pathogens recognize each other rapidly after touching and then trigger the signal pathways respectively to achieve the purpose of disease resistance or infection. While we know that there are interactions between *R* gene and *Avr* gene, the disease resistance mechanisms and a series of signalling pathways remain to be studied.

Research into molecular responses at early infection stage for fungal pathogen and host plant interactions are of significance for pathogenesis study and resistant crop breeding. Understanding of the molecular events occurring at the early interaction stage would be an essential step for describing the initial mechanism of pathogen–host interactions in many important agricultural disease systems and medical pathogen systems. The candidate genes revealed by the study of early interaction could bring out the target genes for crop improvement by transgenic methods or genome editing. The manifest conclusions obtained in this field are still limited. Further investigations and new technologies should be employed in this direction, which will attract more and more researchers to join this creative area and contribute to the agriculture.
